# Risk factors for fracture redisplacement after reduction and cast immobilization of displaced distal radius fractures in children: a meta-analysis

**DOI:** 10.1007/s00068-019-01227-w

**Published:** 2019-09-09

**Authors:** Alysia Sengab, Pieta Krijnen, Inger Birgitta Schipper

**Affiliations:** Department of Trauma Surgery, Leiden University Medical Centre, Post Zone K6-R, P.O. Box 9600, 2300 RC Leiden, The Netherlands

**Keywords:** Radius fracture, Paediatrics, Displacement, Redisplacement, Risk factors, Cast immobilization, Cast index, Three-point index

## Abstract

**Purpose:**

Displaced distal radius fractures in children are common and often reduced if necessary and immobilized in cast. Still, fracture redisplacement frequently occurs. This can be prevented by fixation of fracture fragments with K-wires, but until now, there are no clear guidelines for treatment with primary K-wire fixation. This meta-analysis aimed to identify risk factors for redisplacement after reduction and cast immobilization of displaced distal radius fractures in children, and thereby determine which children will benefit most of primary additional K-wire fixation.

**Methods:**

Eight databases were searched to identify studies and extract data on the incidence of and risk factors for redisplacement of distal radius fractures after initial reduction and cast immobilization in children.

**Results:**

Twelve studies, including 1256 patients, showed that initial complete displacement (odds ratio [OR] 4.69, 95% confidence interval [CI] 2.98–7.39) and presence of a both-bone fracture (OR 1.95, 95% CI 1.34–2.85) were independent risk factors for redisplacement. Anatomical reduction reduced the redisplacement risk (OR 0.14, 95% CI 0.05–0.40). No significant influence on redisplacement risk could be established for female sex, experience level of the attending surgeon, Cast Index < 0.8, Three-Point Index < 0.8 and patient’s age.

**Conclusions:**

For children with a displaced distal radius fracture, the presence of a both-bone fracture, complete displacement of the distal radius and non-anatomical reduction are risk factors for redisplacement after reduction of their initially displaced distal radius fracture. Children with one or more of these risk factors probably benefit most of reduction combined with primary K-wire fixation.

## Introduction

Distal radius fractures account for up to 35% of all paediatric fractures and are mostly caused by a fall on the outstretched hand or direct blow to the arm [1–4]. For substantially displaced paediatric distal radius fractures, fracture reduction and cast immobilization is often the treatment of choice. Recent studies showed, however, that redisplacement rates are considerable and range from 21 to 39% after conservative treatment [5–9]. To prevent redisplacement after reduction, the fracture can be fixated with K-wires. However, this treatment also has disadvantages as it can lead to complications such as pin-tract infection, neuropraxia and premature closure of the physis [10–14]. Therefore, it is important to balance advantages and disadvantages of non-operative and operative treatment in relation to the risk of redisplacement and its effect on final outcome. Many studies have been performed to identify risk factors for redisplacement showing varying results. Several studies recommended additional K-wire fixation for not optimally reduced distal radius fractures, while others recommended additional K-wire fixation for all completely displaced fractures, even after an acceptable closed reduction [7, 9, 15, 16]. More recently, the quality of cast moulding was evaluated as a potential risk factor for redisplacement, however, without univocal results [5, 17–19]. The aim of this meta-analysis was to evaluate the available literature on risk factors for redisplacement of distal radius fractures in children treated with reduction and cast immobilization, and thereby determine which children will benefit the most of primary K-wire fixation additional to cast immobilization. This will aid in establishing guidelines for the treatment of displaced distal radius fractures with primary K-wire fixation.

## Materials and methods

This meta-analysis was performed according to the preferred reporting items for systematic reviews and meta-analyses (PRISMA) guidelines [20].

### Search strategy

A literature search was performed in PubMed, Embase, Web of Science, Cochrane, CENTRAL, CINAHL, Academic Search Premier and Science Direct on April 12th, 2019. The search strategy was composed by an experienced medical librarian. It included different synonyms of the keywords Radius Fractures, Child, Displaced, Casts and Risk Factors.

### Study selection

Articles were selected if they (1) included skeletally immature patients, (2) that had a displaced distal radius fracture (with or without a concomitant distal ulnar fracture) requiring fracture reduction, (3) and were treated with above- or below-elbow cast immobilization. Articles had to be written in English and describe risk factors for redisplacement. Because definitions for displacement and redisplacement vary amongst studies, no predefined definition was applied for study selection, but only studies with commonly used and comparable definitions for (re)displacement (Table 1) were included in the meta-analysis. An additional criterion was that the articles reported odds ratios (ORs) of the risk factors for redisplacement, or provided sufficient information to calculate the ORs. Articles were excluded if these (1) concerned Salter Harris 3 and/or 4 fractures (as these usually require surgical treatment), (2) were case reports, reviews, conference abstracts, letters to the editor or cadaver studies, (3) also analysed other forearm fractures or treatment options, and the results for the displaced distal radius fractures treated with cast immobilization could not be extracted separately, or (4) reported only on potential risk factors that were not reported in one of the other included articles. Reference lists of the potentially relevant full-text articles were searched for additional eligible studies, which were included if the above-mentioned inclusion criteria applied. Study selection, data extraction and assessment of risk of bias were performed by two reviewers (AS and PK). Disagreement was resolved by discussion.

**Table 1:** Characteristics of included studies

Author	Type of study	No. of fractures	Mean age in years (range) or (± SD)	Fracture type	Treatment*	Duration of follow-up	No. of fractures redisplaced (%)	Redisplacement (definition)	Indication for reduction (definition)
Alemdaroglu [19] (2008)	Prospective	75	10.6	Metaphyseal radius and ulnar fractures	1, BEC	4 weeks	17/75 (22.7)	(1) ≥ 10° dorsal/volar angulation, or (2) ≥ 5° radial deviation, or (3) ≥ 3 mm translation, or (4) a combination of ≥ 2 mm translation and ≥ 5°angulation	One of the following: >20°dorsal angulation, > 10° radial deviation, > 4 mm translation. Or combination of at least 2 of the following: > 10° dorsal angulation, > 5° radial deviation, ≥ 3 mm translation
Arora [20] (2018)	Prospective	37	Redisplaced: 8.56 (± 2.70) Not displaced: 9.05 (± 3.21)	Metaphyseal radius ± ulnar fracture	1, AEC	6 weeks	8/37 (21.6)	Bayonet apposition < 1 cm (age < 9 years), angulation up to 30 degrees in sagittal plane (> 5 years of growth remaining), acceptable angulation reduced by 5 degrees for each less year of growth remaining, angulation up to 15 degrees in the frontal plane	
Asadollahi [5] (2015)	Prospective	135	9.9 (3–17)	Distal physeal/metaphyseal radius ± ulnar fracture	2, BEC	8 weeks (redisplaced)	39/135 (28.8)	(1) ≥ 10°dorsal/volar angulation, or (2) ≥ 5° radial deviation, or (3) ≥ 3 mm translation, or (4) combination of ≥ 2 mm translation and ≥ 5° angulation	Based on the age of the patient, age of fracture, location of fracture, presence of clinical deformity, and treating consultant clinical judgment. Some loss of position was accepted with the expectation of satisfactory remodelling
Debnath [25] (2011)	Retrospective	156	9.8 (2–15)	Distal third radius ± ulnar fracture	2, AEC	6 weeks	30/156 (19.2)	Re-angulation > 20° and clinically evident deformity	Re-angulation > 20° and clinically evident deformity
Devalia [26] (2011)	Retrospective	55	Redisplaced 10.8 (4–16.8) Not displaced 12 (6–16.8)	<4 cm of distal radius physis	2, type of cast not reported	‘Until discharge’	14/55 (25.4)	> 10°angulation on lateral radiographs, any angulation on postero-anterior radiograph and loss of more than 50% apposition on either radiographs	
Ghimire [30] (2016)	Prospective	58	Redisplaced: 10.4(± 3.24) Not displaced: 10.68 (± 3.11)	Distal third radius	1, 2, hematoma block or brachial block Not reported	6 weeks	20/58 (34.5)	Translation of > 5 mm in any plane, angulation > 20° in sagittal plane or any deviation > 5° in coronal plane or combination of > 10° angulation in sagittal plane and > 2 mm of translation	
Haddad [31] (1995)	Retrospective	86	9 (4–16)	Closed extra-articular distal forearm fracture	2, not reported	Not reported	18/86 (21)	Angulation > 20° at 1 week	
Jordan [27] (2015)	Retrospective	107	10.0	Distal third radius ± ulna	Not reported	4–6 weeks	29/107 (27)	> 20°angulation or < 50% of bony contact from the normal anatomical position	‘Not standardized’
Pretell [28] (2012)	Retrospective	161	10.2	Distal metaphyseal distal radius ± ulna	2, not reported	2.8 months (0.7–14.5)	57/161 (35)	≥ 15° angulation in coronal plane for all ages and/or angulation in the sagittal plane up to 30° if more than 5 years of growth remaining and 5° less for each year less than five	
Proctor [7] (1993)	Retrospective	68	(1–16)	Distal radius	Not reported, AEC and BEC	Not reported	23/68 (34)	> 20° angulation, or less than 50% apposition of the fragments	
Schneider [29] (2007)	Retrospective	205	10 (3–16)	Epiphyseal (SH1/2), metaphyseal radius ± ulnar fracture	1 or 2, AEC	3 months	47/205 (23)	>20° angulation if < 10 years old and any angulation in older children	
Webb [32] (2006)	Randomized Controlled Trial	113	9.8 (4–16)	Distal third forearm fracture	1 (if not acceptable reposition then 2), AEC or BEC	7.7 months (3.5–11)	11/113 (9.7)	Increase of > 10° angulation or deviation and > 20% displacement compared with the post-reduction values	

*AEC*   above-elbow cast, *BEC*  below-elbow cast

*Closed reduction and cast immobilization under (1) conscious sedation on the ED or (2) general anaesthesia

### Data extraction

**Fig. 1:**
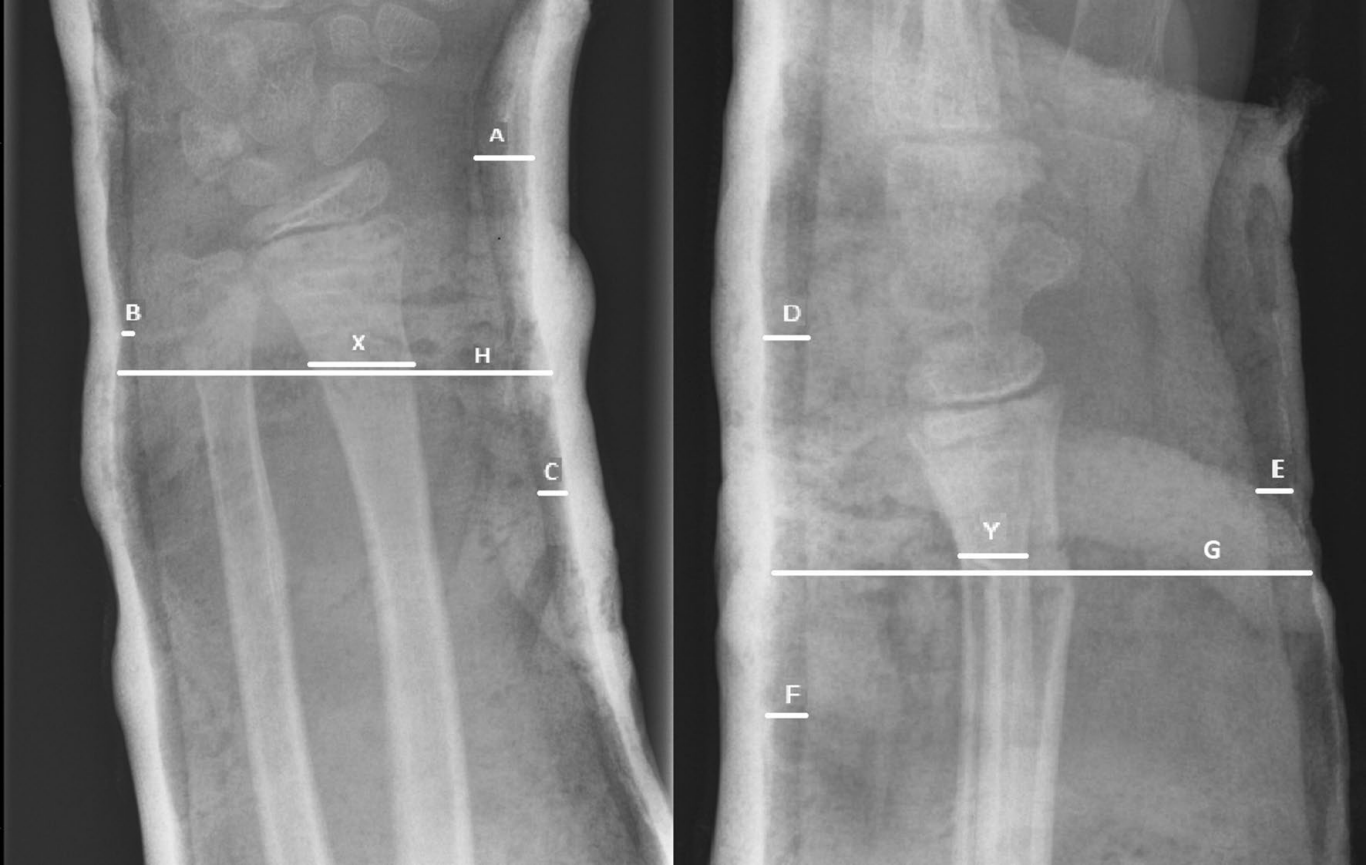
Calculation of the Cast Index and Three-Point Index on the anteroposterior (on the left) and lateral (on the right) radiographs. The Cast Index is defined as the inner cast width at the fracture site on the lateral radiograph (**G**) divided by the inner cast width on the anteroposterior radiograph (**H**). The Three-Point Index is defined as [(A+ B+C)/X] + [D+ E+F)/Y] with on the anteroposterior radiograph: **A** the narrowest radial-side gap between cast and skin around radiocarpal joint or scaphoid; **B** the narrowest ulnar side gap between cast and skin within 1 cm of the fracture; **C** the narrowest radial-side gap, 3–5 cm proximal to the fracture side. On the lateral radiograph, **D** the narrowest dorsal-side gap between skin and cast at radiocarpal joint or proximal carpal row; **E** and **F** similar to **B** and **C,** however, at the volar- and dorsal-side gap, respectively, on the lateral radiograph [5, 18, 19]

From the included articles, data were extracted on study characteristics (author, publication year, type of study), number of included patients, mean age, fracture characteristics (isolated distal radius or both-bone fracture), definition of indications for fracture reduction and redisplacement, type of anaesthesia and treatment (conscious sedation or general anaesthesia, closed reduction and cast immobilization or additional K-wire fixation, above- or below-elbow cast), outcome (redisplacement rate), and risk factors (age, gender, isolated radius fracture or both-bone fracture, complete displacement, quality of reduction, Cast Index, Three-Point Index, surgeon’s level of experience). The definitions for redisplacement and indications for reduction are reported in Table 1. The calculation of the Cast Index and the Three-Point Index is illustrated in Fig. 1. Optimal values for both indexes are considered below 0.8 [5, 18, 19]. A previous meta-analysis, published by Hendrickx et al. and Van den Bekerom et al., showed no significant difference in redisplacement rate after treatment with either above- or below-elbow cast immobilization [21, 22]. Therefore, the type of cast (above or below elbow) was not included in the risk factor analysis.

### Statistical analysis

A meta-analysis using Review Manager 5.3 was performed for the selected studies that applied similar data definitions and had comparable study groups. When available, results from multivariate analysis were used instead of univariate analysis. ORs were pooled using the generic inverse variance. The random-effects model was used for all meta-analysis. Statistical heterogeneity between studies was assumed if *p* < 0.10 for the Cochran’s Chi-square test or I^2^ > 50% [23].

### Risk of bias

Risk of bias in the included studies was assessed according to the ‘Quality in Prognosis Studies’ (QUIPS) tool as low, moderate or high in six domains including study participation, study attrition, prognostic factor measurement, outcome measurement, study confounding and statistical analysis and reporting [24]. Bias due to prognostic factor measurement was scored as moderate if it was not clear who performed the risk factor measurements. Bias due to confounding was scored as low if multivariate analysis was performed and as moderate in case of univariate analysis only (Table 2).

**Table 2:** Quality of included studies according to the QUIPS tool

Author	Risk of bias due to study participation	Risk of bias due to study attrition	Risk of bias due to prognostic factor measurement	Risk of bias due to outcome measurement	Risk of bias due to study confounding	Risk of bias due to analysis
Alemdaroglu [19]	Low	Low	Low	Low	Low	Low
Arora [20]	Low	Low	Moderate	Low	Moderate	Low
Asadollahi [5]	Low	Low	Low	Low	Low	Low
Debnath [25]	Low	Low	Low	Low.	Moderate	Low
Devalia [26]	Low	Low	Low	Low	Moderate	Low
Ghimire [30]	Low	Low	Moderate	Low	Moderate	Low
Haddad [31]	Low	Low	Moderate	Low	Moderate	Moderate
Jordan [27]	Low	Low	Low	Low	Moderate	Low
Pretell Mazzini [28]	Low	Moderate	Low	Low	Low	Low
Proctor [7]	Low	Low	Low	Low	Low	Low
Schneider [29]	Low	Low	Low	Low	Moderate	Low
Webb [32]	Low	Low	Low	Low	Low	Low

## Results

### Literature search

**Fig. 2:**
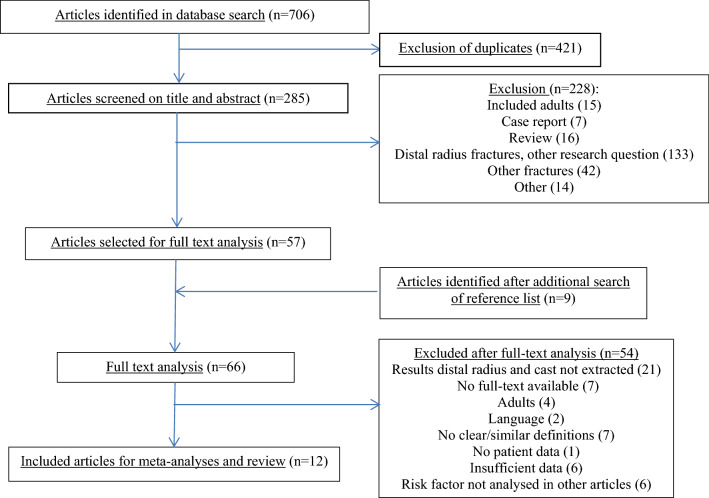
Flowchart of included articles

The electronic search identified a total of 706 potentially eligible articles. After removal of duplicates, 285 articles remained and were screened for eligibility based on title and abstract. Fifty-seven articles were eligible and selected to read the full text. After screening the reference lists of these 57 articles, nine more potentially relevant studies were identified. Twelve articles that met the inclusion criteria and reported on similar age groups and definitions for redisplacement were included in this meta-analysis (Fig. 2) [5, 7, 18, 19, 25–32].

### Study characteristics

Table 1 shows the characteristics of the twelve included studies. The studies were published between 1993 and 2018 and included a total of 1256 patients who received cast immobilization after reduction of a displaced distal radius fracture. Seven studies had a retrospective design, four a prospective design and one RCT was included. Treatment consisted of reduction of the displaced fracture under conscious sedation on the ED, hematoma or brachial block, or general anaesthesia. Immobilization consisted of either an above- or below-elbow cast. The follow-up ranged between 1 and 7.7 months. Definitions for displacement and redisplacement are reported in Table 1 [5, 7, 18, 19, 25–32].

### Outcome

The mean follow-up ranged between 1 and 7.7 months. The overall redisplacement rate after initial reduction ranged from 9.7 to 35% [5, 7, 18, 19, 25–32]. Of all redisplaced fractures, 61% (191/313) received secondary treatment.

### Risk factors

**Fig. 3:**
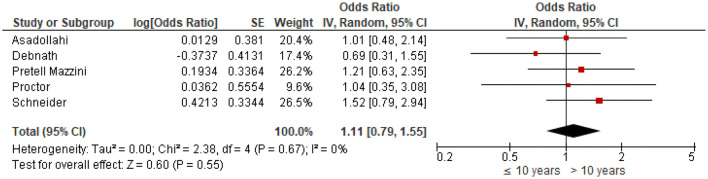
Risk of redisplacement in patients of below 10 years of age versus above 10 years

**Fig. 4:**
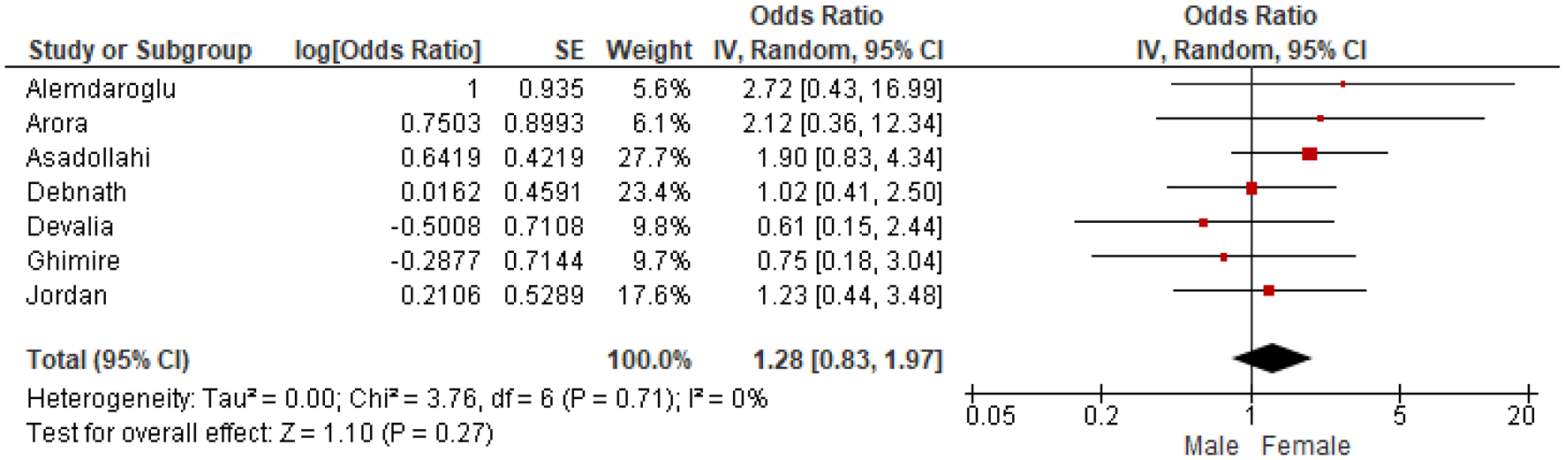
Risk of redisplacement in male versus female patients

**Fig. 5:**
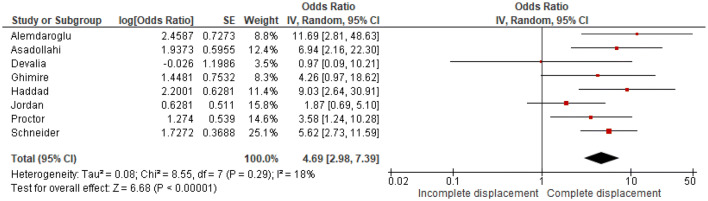
Risk of redisplacement after incomplete versus complete displacement

**Fig. 6:**
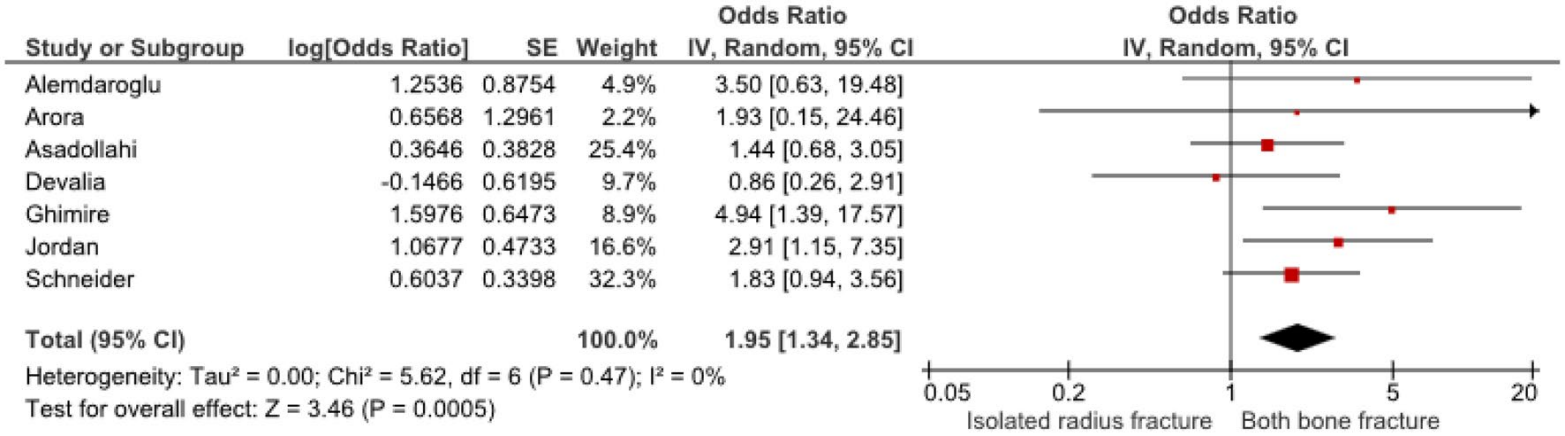
Risk of redisplacement after isolated radius versus a both-bone fracture

**Fig. 7:**
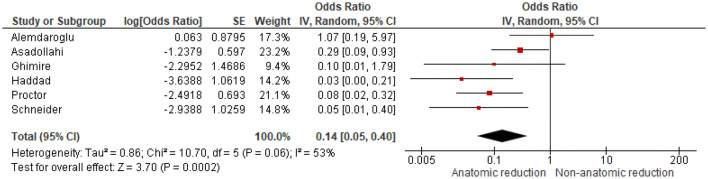
Risk of redisplacement after anatomic reduction versus non-anatomic reduction

**Fig. 8:**
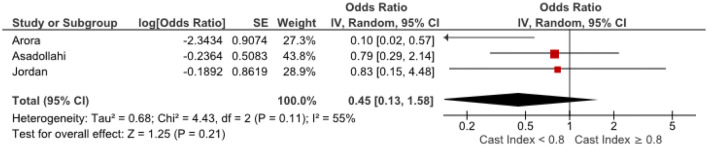
Risk of redisplacement after Cast Index < 0.8 versus Cast Index > 0.8 on post-reduction radiograph

**Fig. 9:**
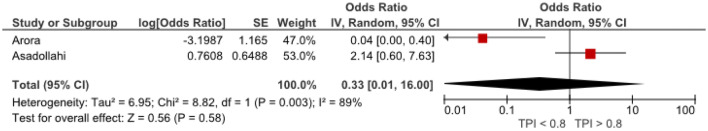
Risk of redisplacement after Three-Point Index < 0.8 versus Three-Point Index > 0.8 on post-reduction radiograph

**Fig. 10:**
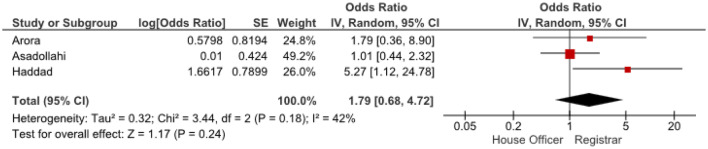
Risk of redisplacement after treatment by a house officer versus registrar

Odds ratios were extracted or calculated from the 12 included studies. If insufficient data were available, corresponding authors were contacted. Asadollahi et al. supplied supplementary data [5]. The ORs were pooled for eight predictors (age, gender, isolated radius of a both-bone fracture, complete displacement, quality of reduction, Cast Index, Three-Point Index and surgeon’s level of experience) for redisplacement in children after reduction of a displaced distal radius fracture. Age < 10 years vs > 10 years (OR 1.11, 95% CI 0.79–1.55) and female sex (OR 1.28, 95% CI 0.83–1.97) were not significant risk factors for redisplacement (Figs. 3, 4). Complete displacement (mostly defined as one shaft width), when compared to incomplete displacement (OR 4.69, 95% CI 2.98–7.39) and a both-bone fracture, when compared to an isolated radius fracture (OR 1.95, 95% CI 1.34–2.85) were significant risk factors for redisplacement (Figs. 5, 6). Anatomic reduction significantly reduced the risk of redisplacement (OR 0.14, 95% CI 0.05–0.40) when compared to non-anatomic reduction (Fig. 7). The Cast Index and Three-Point Index, both with optimal values considered below 0.8, were not predictive for redisplacement (respectively, OR 0.45, 95% CI 0.13–1.58 and OR 0.33, 95% CI 0.01–16) (Figs. 8, 9) [5, 17–19, 27]. However, it should be noted that the results of the studies for both indexes were statistically heterogeneous (I^2^ > 50%). The experience of the trainee/house officer compared to that of a senior registrar/consultant as the treating physician was also not a risk factor for redisplacement (OR 1.79, 95% CI 0.68–4.72) (Fig. 10).

### Risk of bias

The risk of bias of the included studies was low for almost all six domains (Table 2). Arora et al., Haddad et al. and Ghimire et al. scored moderate on the risk of bias in the domain of prognostic factor measurement, because it was not reported who performed the measurements of the potential risk factors for redisplacement [19, 30, 31]. Many of the included studies also scored moderate on biases in the domain of study confounding. This is because only univariate analysis was performed and no multivariate analysis [19, 25, 26, 29–31, 33].

## Discussion

The aim of this meta-analysis was to identify the possible risk factors for redisplacement of distal radius fractures in children after reduction and cast immobilization and thereby determine which children benefit the most from additional K-wire fixation after fracture reduction.

The results show that the presence of a both-bone fracture, initial complete displacement of the distal radius fragment and non-anatomical reduction are significant risk factors for redisplacement and, therefore, present as indications for reduction and additional primary K-wire fixation of paediatric displaced distal radius fractures.

Fracture-related factors are often studied as potential risk factors for redisplacement. Our results showed that it is important to achieve anatomic reduction to diminish the risk of redisplacement (OR 0.14, 95% CI 0.05–0.40) (Fig. 7). One can imagine that achieving anatomical reduction is dependent on multiple other factors such as the experience of the treating physician and available resources (e.g. type of analgesics/sedatives, C-arm fluoroscopy) at the time of reduction. Nevertheless, the experience of the treating physician was not found to influence the risk of redisplacement (Fig. 10). This result is based on only three studies and could potentially be biased, as the trainee is often supervised by the attending senior/consultant. Furthermore, no difference was made between trainees with relatively little experience or multiple years of experience and between (orthopaedic) surgeons and emergency physicians. Despite this potential bias, Proctor et al. and Monga et al. found similar results [7, 34]. One can also imagine that achieving anatomical reduction is more likely to be successful when there is a good understanding of the fracture mechanism; reduction takes place at the operating room, under conscious sedation or general anaesthesia and with the use of C-arm fluoroscopy. Unfortunately, there was insufficient information in the included articles to analyse whether or not these factors (i.e. type of anaesthesia, C-arm fluoroscopy) are of any influence on the risk of redisplacement. Three studies, not included in this meta-analysis, did report on these issues. Bear et al. compared haematoma block analgesia to procedural sedation and found no significant difference in radiographic alignment between groups. Moreover, haematoma block analgesia resulted in similar pain control, shorter duration of stay at the emergency department and comparable patient satisfaction as procedural sedation [35]. Luhmann et al. confirm these results in their study [36]. Lee et al. retrospectively analysed the use of C-arm fluoroscopy and showed that patients undergoing closed reduction with assistance of the mini C-arm fluoroscopy had significant improvement in quality of the reduction (average angulation in degrees ± standard deviation; 6 ± 4 vs. 8 ± 6; *p* = 0.02), less second reduction attempts and less need for operative treatment (2/113 vs. 14/166, *p* < 0.0001) compared to reduction without the use of the C-arm fluoroscopy [37].

In 1994, Chess et al. introduced the Cast Index as an indicator for the quality of cast moulding. They described the quality of cast moulding as a risk factor for redisplacement after reduction of a paediatric displaced distal radius fracture [17]. Since then, more studies have been published about the quality of cast moulding and several other cast-related indices such as the Three-Point Index, Gap Index and Padding Index were analysed [5, 18, 19, 26, 38]. Unfortunately, the studies reporting the risk of redisplacement for all the different cast-related indices could not be combined in this meta-analysis since these indices provide heterogeneous outcomes. Based on the results of the three included papers that address this topic, the Cast Index and Three-Point Index do not seem to predict redisplacement after the first reduction in displaced distal radius fractures in children (Figs. 8, 9) [5, 15, 19]. More homogeneous studies are needed, however, to draw firm conclusions regarding the predictive value of cast-related indices since this potential risk factor can be positively influenced with little effort. After reduction and application of the cast, measurements on plain radiographs can be made and if needed, and the cast can be adjusted to reduce the risk of redisplacement. Even though the Cast Index is easier to measure, the Three-Point Index was found to be superior in predicting redisplacement when compared to the Cast Index (sensitivity 94.7%, specificity 95.2%, NPV 98.4%, PPV 85.7% for Three-Point Index and sensitivity 63.2%, specificity 52.4%, NPV 82.5%, PPV 28.6% for Cast Index) with high inter- and intra-observer reliability (intra class correlation coefficient 0.99) [18, 19]. The studies included in the present meta-analysis reported redisplacement rates between 9.7% and 35% after reduction and cast immobilization of displaced distal radius fractures in children. Only 61% of the 313 redisplaced fractures received secondary treatment: 38 received repeat reduction and cast immobilization, 128 had additional K-wire fixation after repeat reduction, 10 received ORIF, 1 received plate fixation and 3 patients received external fixation. Eighteen patients were reported to have had ‘surgery, CRIF or ORIF’ and for three patients, the cast was wedged as a secondary treatment. Fifty-eight (19.0%) patients were considered to have enough potential for remodelling and received no further treatment after redisplacement. For the remaining 20.0% with a redisplaced fracture, it was not explicitly reported why secondary treatment was not deemed necessary. A reason might be that the definitions for redisplacement and the indications for secondary treatment were not similar in all studies. Also, wait and see policies are probably also based on the expectation that there is sufficient growth and the remodelling potential in the injured bone in children. Finally, the fact that an association of repeat reduction with growth disturbances and worse functional outcome has been described may have contributed to a reserved attitude towards repetitive reduction [39, 40].

This meta-analysis has several limitations. Although many studies have reported on the risk factors for redisplacement, only a few used similar indications for fracture reduction. This is partly due to the absence of globally accepted criteria for when to reduce a paediatric distal radius fracture. For all of the included articles, the criteria for redisplacement were angulation of at least 10 degrees, more than 2 mm translation or more than 20% of displacement when compared to post-reduction values. Furthermore, in current decision-making on fracture reduction in children, the potential for remodelling in relation to the acceptable amount of displacement is not adequately incorporated. This is also shown in the included articles, as only three out of twelve reported on criteria for reduction that were specified by the age of the patient and thereby the expected remodelling potential (Table 1). Younger children have a greater spontaneous remodelling potential and, therefore, for them, larger amounts of displacement can be accepted without reduction and immobilization (cast and/or K-wire fixation). In practice, this should be considered not only before primary reduction but also if redisplacement occurs. However, including this information in our analysis would be difficult since remodelling not only depends on age but also, for example, on race and sex. Despite helpful AO guidelines, the definitions for redisplacement, indications for fracture reduction and inadequate incorporation of the potential for remodelling differed between the studies. This could potentially lead to over- or underestimation of the true redisplacement risk when applying our results to future patients.

No statistical heterogeneity in study results for the risk factors was found, except for analysis of the Cast Index, Three-Point Index and quality of reduction (I^2^ > 50%).

A third limitation is the heterogeneous presentation of other potential predictors for redisplacement. This includes several cast-related indices, comminution of the fracture and distance of the fracture to the physis that could be related to redisplacement after reduction of a fracture. Unfortunately, due to the heterogeneous data, these potential predictors could not be included in this meta-analysis.

This meta-analysis shows that for children with a displaced distal radius fracture, the presence of a both-bone fracture, complete displacement of the distal radius and non-anatomical fracture reduction are risk factors for redisplacement of their initially displaced distal radius fracture. Children with one or more of these risk factors will probably benefit most of the reduction combined with primary K-wire fixation.
